# Prediction of virus-host infectious association by supervised learning methods

**DOI:** 10.1186/s12859-017-1473-7

**Published:** 2017-03-14

**Authors:** Mengge Zhang, Lianping Yang, Jie Ren, Nathan A. Ahlgren, Jed A. Fuhrman, Fengzhu Sun

**Affiliations:** 1 Molecular and Computational Biology Program, University of Southern California, Los Angeles, California USA; 2 Department of Biological Sciences and Wrigley Institute for Environmental Studies, University of Southern California, Los Angeles, California USA; 3 College of Sciences, Northeastern University, Shenyang, China; 4 Centre for Computational Systems Biology, School of Mathematical Sciences, Fudan University, Shanhai, China; 5 Biology Department, Clark University, Worcester, Massachusetts USA

## Abstract

**Background:**

The study of virus-host infectious association is important for understanding the functions and dynamics of microbial communities. Both cellular and fractionated viral metagenomic data generate a large number of viral contigs with missing host information. Although relative simple methods based on the similarity between the word frequency vectors of viruses and bacterial hosts have been developed to study virus-host associations, the problem is significantly understudied. We hypothesize that machine learning methods based on word frequencies can be efficiently used to study virus-host infectious associations.

**Methods:**

We investigate four different representations of word frequencies of viral sequences including the relative word frequency and three normalized word frequencies by subtracting the number of expected from the observed word counts. We also study five machine learning methods including logistic regression, support vector machine, random forest, Gaussian naive Bayes and Bernoulli naive Bayes for separating infectious from non-infectious viruses for nine bacterial host genera with at least 45 infecting viruses. Area under the receiver operating characteristic curve (AUC) is used to compare the performance of different machine learning method and feature combinations. We then evaluate the performance of the best method for the identification of the hosts of contigs in metagenomic studies. We also develop a maximum likelihood method to estimate the fraction of true infectious viruses for a given host in viral tagging experiments.

**Results:**

Based on nine bacterial host genera with at least 45 infectious viruses, we show that random forest together with the relative word frequency vector performs the best in identifying viruses infecting particular hosts. For all the nine host genera, the AUC is over 0.85 and for five of them, the AUC is higher than 0.98 when the word size is 6 indicating the high accuracy of using machine learning approaches for identifying viruses infecting particular hosts. We also show that our method can predict the hosts of viral contigs of length at least 1kbps in metagenomic studies with high accuracy. The random forest together with word frequency vector outperforms current available methods based on Manhattan and $d_{2}^{*}$ dissimilarity measures. Based on word frequencies, we estimate that about 95% of the identified T4-like viruses in viral tagging experiment infect Synechococcus, while only about 29% of the identified non-T4-like viruses and 30% of the contigs in the study potentially infect Synechococcus.

**Conclusions:**

The random forest machine learning method together with the relative word frequencies as features of viruses can be used to predict viruses and viral contigs for specific bacterial hosts. The maximum likelihood approach can be used to estimate the fraction of true infectious associated viruses in viral tagging experiments.

**Electronic supplementary material:**

The online version of this article (doi:10.1186/s12859-017-1473-7) contains supplementary material, which is available to authorized users.

## Background

Viruses are the most abundant organism on earth with the number of viruses over 10-fold higher than the number of bacteria [1, 2]. Viruses play important roles in almost all domains of life due to their wide distribution in both the environment and the body of living organisms [3, 4] including water [5, 6], soil and the human body [3, 7]. To produce progeny, viral particles must infect a living organism, namely, the hosts, by first infecting the host cell and later hijacking the host cellular replication mechanisms. Bacteria, archaea and animals are the natural virus hosts. Viral infections often cause cellular and physiological changes in the host cells, for example, altering the genomic sequences of their hosts [8], and sometimes causing dysfunctions in the hosts [9–12].

The class of viruses that specifically infect bacteria is known as bacteriophages. They are of special interest to ecologists and microbiologists because of the close connection that bacteria have with the human health and the environment. For example, the human microbiomes can be affected by bacteriophages [13]. Some bacteriophages have been shown to alter the composition of microbial communities leading to changes in these communities.

Despite the importance of viruses in microbial communities, the mechanisms of viruses infecting hosts are not fully understood. Metagenomic studies using next generation sequencing (NGS) technologies such as the Human Microbiome Project (HMP) [14, 15] and the global ocean survey (GOS) [16] generated a large number of short read data targeting total genomic (cellular) or fractionated virus particles. Many viral sequences are generated without knowing hosts. This opens up an opportunity for the study of virus-host association by utilizing this wealth of sequencing information. Thus, the primary objective of this study is the development of computational approaches for the prediction of infectious associations between viruses and given prokaryotic hosts.

Although this problem has not been heavily investigated before, we are aware of two relevant studies [17, 18]. Ahmed et al. [18] developed a computational method based on “oligostickiness” for studying virus-host infectious association relationship. However, the authors based their studies on only 25 viruses and 7 bacterial hosts and the software is not available (per communications with the authors). Roux et al. [17] used Manhattan distance between the frequency vectors of word patterns (*k*-tuple, gram) for a virus and a potential bacterial host to study their relationships and some promising results were obtained. The study showed that the word frequency vectors of viruses contain information about their hosts. Based on this study, we hypothesize that machine learning methods based on the word frequency vectors of viruses can be used to predict virus-host infectious associations more accurately.

In this study, we collected 1,426 completely sequenced viral genomes with precisely identified hosts from the NCBI phage genome database. Among all the bacteria at the genus level, we focus on 9 bacterial genera each of which containing at least 45 viruses infecting the hosts, providing large sample sizes to optimize the machine learning methods. Together they have been identified as the hosts of 836 out of 1,426 viral genomes (Additional file 1: Table S1). In addition, most of these 9 hosts have been shown to play important roles in microbiome studies and they are also closely related to human diseases [19–24]. Therefore, identification of viral sequences that infect each of the 9 hosts from the vast amount of newly generated viral sequence data has high significance.

It has been hypothesized and data has shown that the word pattern usage between viruses and their hosts tend to be more similar than those for random virus-host pairs [17]. This hypothesis is based on the fact that the virus is dependent on the molecular machinery of its host to replicate, so the virus is expected to adopt similar word pattern usage of the host, evolving to maximize replication. Therefore, infectious virus-host pairs have similar word pattern usages. Thus, we decided to represent each virus by a feature vector based on the word pattern usage. However, it is not clear what the best feature vector representation should be. In this study, we study four different feature vector representations based on the counts of word patterns.

Then we investigated different supervised learning methods based on these feature vectors to predict viruses that potentially infect a particular host. In this study, we studied the supervised learning methods including logistic regression, SVM, random forest, Gaussian naive Bayes and Bernoulli naive Bayes [25]. We next build frameworks for all of the feature-method combinations. By applying the frameworks on the viral complete genome sequence data of the nine main host genera, we identified the best feature-method combination based on the area under the receiver operating characteristic curve (AUC) scores (Supplementary Methods in Additional file 1). We also studied the effect of word length and genome sequence length on the accuracy of the prediction methods.

New technologies such as viral tagging [26] have been developed to associate viruses with particular hosts. Like all high-throughput biotechnologies, there are many potential false positives (observed associations that are not due to infection) and false negatives (associations missed by the experiments). It is important to estimate the fraction of true infectious associations among observed associations, and to separate true infectious associations from false ones. We applied our approach to estimate the fraction of true infectious associations among observed virus-host associations from viral tagging experiment data [26].

## Methods

### Data description

We downloaded 1426 complete viral genome sequences with known host information from the NCBI viral genome database. The NCBI viral data file contains the genome sequence, the host of the virus, and the year it was identified. We focused on 9 bacterial host genera that have at least 45 infectious viruses identified so that enough data are available for learning. Additional file 1: Table S1 shows the number of knowing infectious viruses identified up to each year from 2010 to 2015 for the 9 bacterial genera.

For each of the 9 bacterial host genera, we built a model to predict new viruses that are potentially capable of infecting the corresponding host. In order to evaluate the performance of a supervised learning method, we needed to partition the data into training data and testing data. Instead of using cross-validation as in most studies, we designed a more realistic scenario to predict future new discoveries of viruses infecting the host given previously knowing infectious viruses. Table 1 shows the positive training data and positive testing data as before and after the chosen cutting year, respectively. The negative training data and the negative testing data were chosen randomly without overlaps from the viruses that were not identified to infect the corresponding host. The sizes of positive training/testing and negative training/testing data were set equal. Besides, in order to reduce the variation of performance introduced by selecting negative training data and negative testing data, we selected the negative data randomly for 50 times. The performance of any method was measured as the average performance over 50 repeats with different negative training and testing data.

**Table 1:** Description of the training and testing data

Bacterial	Cutting	# of viruses	# of viruses	# of
genus	year	before	after the	non-infectious
		cutting year	cutting year	viruses
Bacillus	2012	31	31	1364
Escherichia	2012	141	32	1253
Lactococcus	2013	49	6	1371
Mycobacterium	2013	172	46	1208
Pseudomonas	2013	68	28	1330
Salmonella	2012	32	22	1372
Staphylococcus	2012	43	20	1363
Synechococcus	2012	30	17	1379
Vibrio	2012	39	29	1358

For a specific year, the positive training data set contains viruses infecting the corresponding host identified before the specific year and the positive testing data set contains viruses infecting the corresponding host discovered after the specific year. The negative training data and the negative testing data were chosen randomly without overlaps from the viruses that were not identified to infect the host

In addition to identifying the optimal machine learning methods for predicting virus-host infectious associations, we also applied our best prediction method to estimate the fraction of true infectious associations (reliability) in viral tagging experiments [26, 27]. Viral tagging is a new high throughput experimental procedure for detecting viruses infecting a particular host.

In viral tagging experiment, a particular bacterial host of interest is used as bait to fish out viruses potentially infecting the host. The viral sequences are then sequenced using NGS. In the viral tagging experiment of Deng et al. [26], 30 cyanobacterial viral genomes from the assembled reads screened by viral tagging against a particular host Synechococcus sp. WH7803 were obtained. Nineteen out of 30 candidate viral genomes were shown to be T4-like viruses of Synechococcus, and 11 of 30 viral genomes are from non-T4-like viral population. The sequence lengths of the 30 genomes range from 31.5 to 197 kbps with the average length of about 83 kbps.

In addition to the 30 almost complete viral genomes, Deng et al. [26] also generated about 10,864 raw viral short reads with lengths ranging from 15 bp to 580 bp, and the average length of the short reads is 183 bp. We assembled these reads into contigs using the state of art assembly program metaSPAde [28] and we concentrated on 1661 contigs with lengths at least 1.5 kbps.

### Feature definitions

We considered four different definitions of features. For each viral sequence, we counted the number of occurrences *N*_**w**_ for every word of length *k*, $\mathbf {w} \in \mathcal {A}^{k}$, where $\mathcal {A}$ is the set of the alphabet. For example, if we consider DNA sequences and *k*=2, then $\mathcal {A}^{k} = \{AA, AC, AG, AT, CA, \cdots, TT\}$. The four features are defined as follows:

$$\begin{array}{@{}rcl@{}} {{}{\begin{aligned} \mathcal{F}_{1} = \left \{\frac{N_{\mathbf{w}}}{L - k + 1}, \boldsymbol{w} \in \mathcal{A}^{k} \right \}, &~~~~& \mathcal{F}_{2} = \left \{\frac{N_{\mathbf{w}} - E(N_{\mathbf{w}})}{E(N_{\mathbf{w}})}, \boldsymbol{w} \in \mathcal{A}^{k} \right \}, \\ \mathcal{F}_{3} = \left \{\frac{N_{\mathbf{w}} - E(N_{\mathbf{w}})}{\sqrt{E(N_{\mathbf{w}})}}, \boldsymbol{w} \in \mathcal{A}^{k} \right \}, &~~~~& \mathcal{F}_{4} = \left \{\frac{N_{\mathbf{w}} - E(N_{\mathbf{w}})}{\sigma(N_{\mathbf{w}})}, \boldsymbol{w} \in \mathcal{A}^{k} \right \}. \end{aligned}}} \end{array} $$

where *L* is the length of the viral genome sequence; *k* is the length of the words; and *E*(*N*_**w**_) and *σ*(*N*_**w**_) are the expectation and standard deviation of *N*_**w**_ under a certain random model of the viral sequence. Ren et al. [29] proposed to use Markov chains (MC) to model genome sequences and showed promising results for alignment-free genome sequence comparison. In this study, we considered four different models of the viral sequences, including the independent and identically distributed (*i*.*i*.*d*.) model (0-th order MC), 1^*st*^, 2^*nd*^ and 3^*rd*^ order MCs. For each MC model, the probability transition matrix was calculated based on each virus’s own genome sequence, and the resulting *E*(*N*_**w**_) and *σ*(*N*_**w**_) were calculated using the formulas in [30].

The first feature $\mathcal {F}_{1}$ is the standard word frequency vector. The ideas of defining features $\mathcal {F}_{2}, \mathcal {F}_{3}$ and $\mathcal {F}_{4}$ came from the recent studies on alignment-free sequence comparison [29, 31, 32] showing that subtracting the expected word counts from the observed word counts can improve the efficiency of sequence comparison. These feature definitions differ in the denominator for normalizing the word counts. The second feature definition is based on the statistic in the CVtree from Hao’s group [33]. The third and fourth feature definitions are based on the $d^{*}_{2}$ statistic in [31, 32].

### Supervised learning methods

For a given bacterial host, suppose that there are *n* viruses {*V*_1_,*V*_2_,⋯*V*_*n*_} infecting the host and *m* viruses {*V*_*n*+1_,*V*_*n*+2_,⋯*V*_*n*+*m*_} not infecting the host. For a given feature definition, let *x*_*i*_ be the feature vector for the *i*-th virus, *i*=1,2,⋯,*n*+*m* and *y*_*i*_=1 for *i*∈{1,2,⋯,*n*} and *y*_*i*_=0 for *i*∈{*n*+1,*n*+2,⋯,*n*+*m*}. We investigated the following machine learning methods for distinguishing infecting and non-infecting viruses for a particular host. These methods can be found in [25] and we outline them below. Details of these methods can be found in Additional file 1.

#### Logistic regression

Logistic Regression [34] is a commonly used supervised learning approach to predict binary-valued labels. For any virus that we try to predict its infectious association with the given bacterial host, let *Y* be the binary label of the virus and **x** be the feature vector of the virus. In logistic regression, define $h_{\beta }(\mathbf {x}) = \frac {\exp (\beta ^{T} \mathbf {x})}{1+\exp (\beta ^{T} \mathbf {x})}$, where the superscript “*T*” indicates the transpose and *β*=(*β*_1_,*β*_2_,⋯,*β*_*p*_)^*T*^.

We assume that the class label of a given virus with feature vector **x** follows the distribution:

$${{} {\begin{aligned} &\left\{\begin{array}{lll} p(Y = 1 | \mathbf{x}; \beta) = h_{\beta}(\mathbf{x}) \\ p(Y = 0 | \mathbf{x}; \beta)= 1 - h_{\beta}(\mathbf{x})\\ \end{array} \right. \\&\quad \implies\qquad p(Y = y | \mathbf{x}; \beta) = (h_{\beta}(\mathbf{x}))^{y}(1-h_{\beta}(\mathbf{x}))^{1-y} \end{aligned}}} $$

When estimating the parameter *β* with maximum likelihood estimation, in order to deal with the sparsity issue of the data, we added the LASSO regularization (least absolute shrinkage and selection operator) [35] and performed the feature selection with *L*_1_-norm penalization. Then the problem formulation becomes finding *β* such that

$$-\sum_{i=1}^{n+m}\log(p(Y = y_{i} | \mathbf{x}_{i}; \beta))+\lambda\sum_{i=1}^{p}|\beta_{i}| $$

is minimized, where *λ* acts as the penalty term for the number of parameters. In our study, the *λ* is set as the default value, which is 1, in the scikit-learn package [36].

After solving *β*, for a new virus with feature vector **x**, the prediction score is then given by $\hat {y} = h_{\beta }(\mathbf {x})$.

#### Support vector machine with RBF kernel

The support vector machine (SVM) [37] is a popular method for binary classification and it has been successfully applied to many different problems. In general, SVM aims to find the optimal hyperplane that separates the data labeled with *y*_*i*_=1 from the data labeled with *y*_*i*_=0. SVM can be expressed as the following optimization problem

$${{} {\begin{aligned} \min||\mathbf{w}||^{2} \quad \text{subject to} \quad \left\{\begin{array}{ll} \mathbf{w}\cdot\Phi(\mathbf{x}_{i})+b \geq 1 & \quad \text{if}~ \mathnormal{y}_{i} = 1 \\ \mathbf{w}\cdot\Phi(\mathbf{x}_{i})+b \leq 0 & \quad \text{if}~ \mathnormal{y}_{i} = 0\\ \end{array}\right. \end{aligned}}} $$

In our study, we used the Gaussian radial basis function (RBF) as the kernel. Mathematically, the RBF kernel is represented as *K*_*RBF*_(**x**_*i*_,**x**_*j*_)= exp(−*γ*·||**x**_*i*_−**x**_*j*_||^2^)=*Φ*(**x**_*i*_)·*Φ*(**x**_*j*_). As a free parameter of the RBF, a small *γ* represents the data as Gaussian distribution with large variance. Naturally, feature vectors with high dimension will have high variation, so here we set the *γ* as the reciprocal of the dimension of the features [38].

After **w** and *b* are solved, for the feature vector **x** corresponding to any new virus, the prediction score is then determined by $\hat {y} = \mathbf {w}\cdot \Phi (\mathbf {x})+b$.

#### Random forest

Random forest (RF) is a classification method that uses the ensembled classification trees [39], with each tree constructed using a bootstrap sample of the data. At each split, the subtree represents a random subset of the variables. Each tree in the RF is allowed to grow fully to reduce bias in the decision process, while the randomness of variable selection reduces correlation of the individual trees. Therefore, a decision made in the RF is an ensemble that has low bias and low variation, because the decision is made by a collective of low-bias and low correlated trees (see Additional file 1).

#### Naive Bayes

For any virus, let *Y* be the binary label and **x** be the feature vector of the virus. From the Bayes theorem, we have

$$\begin{aligned} &p(\mathnormal{Y}|\mathbf{x}) = \frac{p(\mathnormal{Y})p(\mathbf{x}|\mathnormal{Y})}{p(\mathbf{x})} \quad \implies\\& \qquad \left\{\begin{array}{l} p(\mathnormal{Y}=1|\mathbf{x}) = \alpha \cdot p(\mathnormal{Y}=1)p(\mathbf{x}|\mathnormal{Y}=1) \\ p(\mathnormal{Y}=0|\mathbf{x}) = \alpha \cdot p(\mathnormal{Y}=0)p(\mathbf{x}|\mathnormal{Y}=1) \\ \end{array}\right. \end{aligned} $$

where $\alpha = \frac {1}{p(\mathbf {x})}$. The prediction score is then given by

$$\hat{y} \,=\, \alpha \cdot p(\mathnormal{Y}=1)p(\mathbf{x}|\mathnormal{Y}=1) \,=\, 1-\alpha \cdot p(\mathnormal{Y}=0)p(\mathbf{x}|\mathnormal{Y}=0) $$

Depending on the different assumed distributions of *p*(**x**|*Y*), the naive Bayes [40, 41] method can be further divided into Gaussian naive Bayes and Bernoulli naive Bayes (see Additional file 1).

### Evaluation criteria

For each of the supervised learning methods with any of the four defined features, we trained the model based purely on the training data and obtained a score function. Then we applied the model to the testing data and calculated the prediction scores for each virus. For testing data, a higher prediction score indicates higher probability that the virus infects the host. Based on the prediction scores of the testing data, we calculated the AUC scores [42] (see Additional file 1) as the evaluation criterion for each of the feature-method combinations.

### Estimating the fraction of viruses infecting a host in viral tagging experiments

We assumed that viral contigs derived from viral tagging experiments are a mixture of contigs from viruses infecting the host and those not infecting the host. For an optimal learning method built from the training data, we assumed that the distribution of the scores for viruses not infecting the host follows a beta distribution *Φ*_0_(*α*_0_,*β*_0_) based on our preliminary exploration of the data. Similarly, we assumed that the scores of viruses infecting the host follow another beta distribution *Φ*_1_(*α*_1_,*β*_1_) from the preliminary studies. The distribution of the scores for the observed viral contigs is a mixture of *Φ*_0_(*α*_0_,*β*_0_) and *Φ*_1_(*α*_1_,*β*_1_). Let **P**_obs_(·) denote the distribution for the scores of the viral contigs from the experiments. Then

$$\mathbf{P}_{\text{obs}}(\cdot)= (1-\gamma) \cdot \Phi_{0}(\alpha_{0}, \beta_{0}) + \gamma \cdot \Phi_{1}(\alpha_{1}, \beta_{1}). $$

where *γ* is the fraction of contigs derived from viral sequences infecting the host. We first estimated the parameters (*α*_0_,*β*_0_) and (*α*_1_,*β*_1_) using the moment estimators from the scores for the negative and positive testing data, respectively. Then we used the maximum likelihood approach to estimate the fraction *γ* and its confidence interval.

## Results

### Comparison of different supervised learning methods

**Fig. 1:**
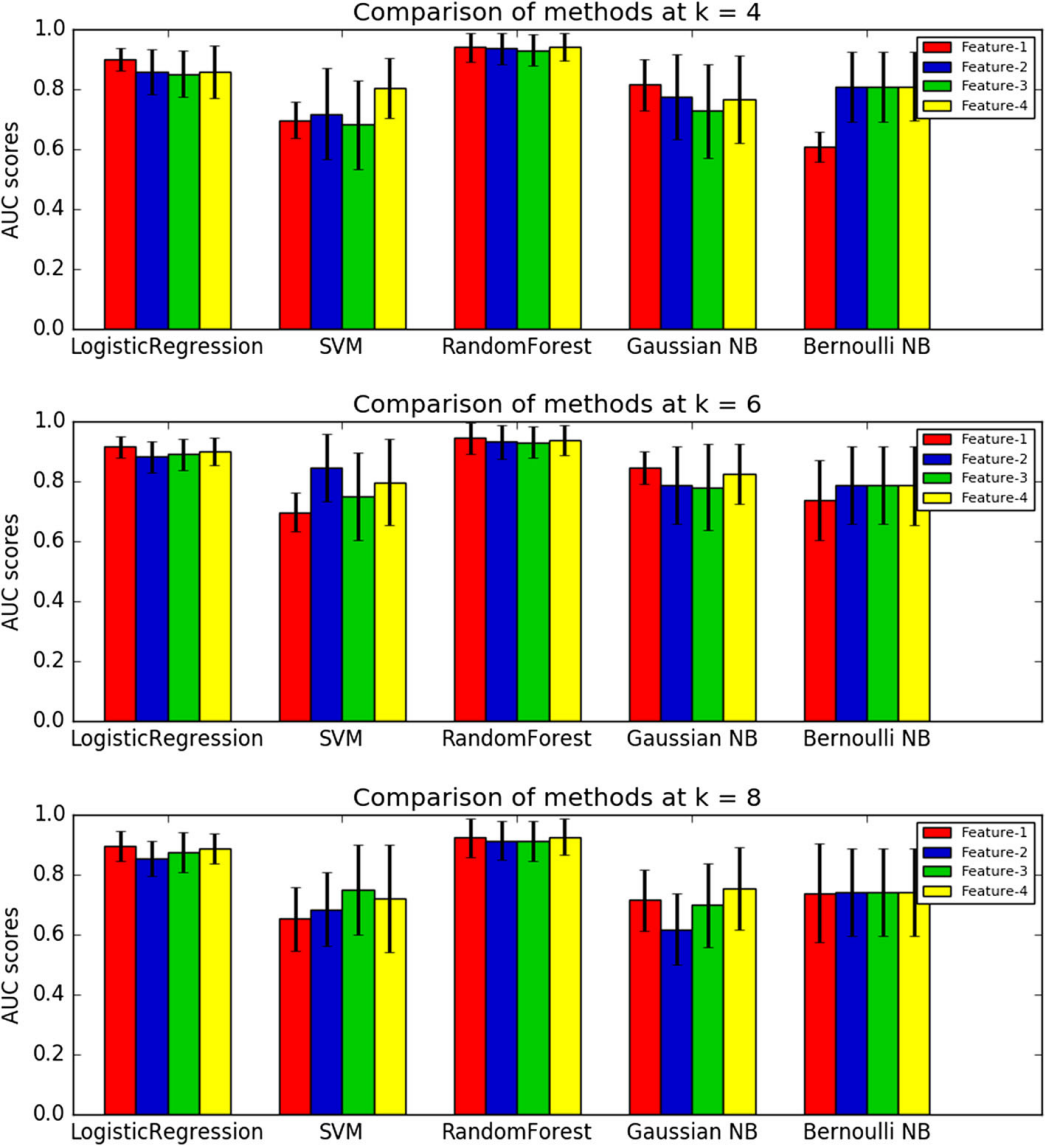
The average AUC scores of the different machine learning methods and features. The averaged AUC scores are calculated by the average of the AUC scores across different hosts and different genome background distributions. The figures from top to bottom are the performances of different word lengths *k*=4, *k*=6 and *k*=8. The black segments on top of the bars are the standard deviation of the AUC scores across different hosts and different genome background distributions

The complete results on the performance of different combinations of features and machine learning methods are given in Additional file 1: Table S2. To see which machine learning method performs the best for a given feature definition, we calculated the average AUC score across the 9 bacterial host genera as well as different background sequence models. The results are shown in Fig. 1. It can be seen from the figure that for all the four features, the RF method outperforms others in general, although there are some exceptions. If we fix the RF method, there are not much performance differences using the four features. Since the first feature definition is the simplest and does not need background models for the sequences, we suggest the use of RF method based on the relative frequencies of word patterns.

An important problem in using word patterns is the determination of the length of word patterns. If the length of word patterns is too short, the frequency vectors can not fully capture the information in the viral sequences. On the other hand, if the length of word patterns is too large, the frequency vector has high variation. Therefore, appropriate choice of the length of word patterns is essential. Fixing the first feature and the RF method, the AUC scores with different word lengths for the 9 host genera are shown in Table 2. When *k*=4, five out of the 9 host genera can achieve AUC over 0.95, one with AUC between 0.90 to 0.95, and three with AUC between 0.85 and 0.90. The average AUC is slightly increased for 6 out of the 9 host genera when *k* is increased from 4 to 6. However, when *k* is increased to 8, the average AUC is significantly decreased for some of the host genera.

**Table 2:** The AUC scores of using RF combined with the first feature with different word pattern lengths across 9 different hosts

	*k*=4	*k*=6	*k*=8
Bacillus	0.856	**0.863**	0.823
Escherichia	**0.878**	0.858	0.807
Lactococcus	0.972	**1.000**	0.988
Mycobacterium	**0.987**	0.985	0.984
Pseudomonas	0.978	**0.981**	0.967
Salmonella	0.889	**0.896**	0.891
Staphylococcus	**0.993**	0.987	0.983
Synechococcus	0.965	**0.978**	0.955
Vibrio	0.936	**0.940**	0.892

The highest score for each host is highlighted in bold

### Potential explanations for the performance variation across different host genera

We are interested in understanding the underlying reasons for the performance variation across the different host genera. We hypothesized that the viral sequences for the host genera with high prediction accuracy are more similar to each other than those for the other host genera. To test this hypothesis, we first calculated the Manhattan distances of the first feature vectors for pairs of viruses infecting each host genus and they are shown in Table 3. Significant associations between the AUC scores and the average Manhattan distances within a group were observed except when *k*=4 (Spearman correlation -0.450 (*p*-value = 0.22), -0.800 (*p*-value = 0.01), and -0.683 (*p*-value = 0.04), for *k*=4,6, and 8, respectively.) This observation indicates that the prediction performance can be partially explained by the average distances among the viruses infecting a host genus.

**Table 3:** Average Manhattan distances of word relative frequency vectors between pairs of viruses infecting each host

	*k*=4	*k*=6	*k*=8
Bacillus	0.342	0.558	1.146
Escherichia	0.372	0.706	1.416
Lactococcus	0.194	0.417	1.020
Mycobacterium	0.292	0.513	1.044
Pseudomonas	0.379	0.633	1.241
Salmonella	0.324	0.568	1.249
Staphylococcus	0.266	0.455	0.984
Synechococcus	0.335	0.516	0.986
Vibrio	0.371	0.658	1.360

We next explored the taxonomic compositions of the viral sequences infecting each host genus. Most viruses in our data belong to the order of Caudovirales that is composed of three major groups: the myoviruses, podoviruses, and siphoviruses (Table 4). These groups of viruses exhibit different host ranges. Myoviruses often have the broadest host ranges and podoviruses and siphoviruses typically have relatively narrow host ranges [43, 44]. Table 4 shows that viruses infecting the nine host genera have very different taxonomic profiles. Viruses infecting Lactococcus and Mycobacterium are primarily siphoviruses. Most of the viruses infecting Staphylococcus belong to either myoviruses or siphoviruses. The three host genera, Lactococcus, Mycobacterium and Staphylococcus, have very high AUC scores over 0.98 when *k*=6. The viruses infecting Synechococcus are primarily myoviruses and the AUC corresponding to this host genus is also high (0.978 when *k*=6). The only exception is the Pseudomonas genus that the viruses infecting the host spread across the three groups while still keeping a high AUC score of 0.981 when *k*=6. For the other host genera, the viruses infecting them generally belong to all three groups and they have relatively low, although decent, AUC scores. We also calculated the entropy of the viruses according to the different groups of viruses for each host and found that the entropy was also highly associated with the AUC scores (Spearman correlation coefficients between entropy and AUC scores are -0.600 (*p*-value = 0.09), -0.750 (*p*-value = 0.02), -0.783 (*p*-value = 0.01) for *k*=4,6, and 8, respectively.)

**Table 4:** The distribution of viruses among three major viral families, Myoviridae, Podoviridae and Siphoviridae, for viruses infecting each of the nine host genera

	Myoviruses	Podoviruses	Siphoviruses	other	*Entropy*
Bacillus	21	8	27	3	1.656
Escherichia	49	30	43	51	1.972
Lactococcus	0	2	53	0	0.472
Mycobacterium	10	0	206	2	0.384
Pseudomonas	38	29	21	8	1.829
Salmonella	10	19	21	4	1.789
Staphylococcus	19	4	35	5	1.535
Synechococcus	28	6	5	8	1.603
Vibrio	20	21	6	21	1.875

The last column is the entropy of the distribution

### Comparison between RF, Manhattan and $d_{2}^{*}$ dissimilarity measures

Roux et al. [17] used Manhattan distance between the word frequency vectors of viruses and bacterial hosts to predict virus-host infectious association. In addition, the $d^{*}_{2}$ statistic [31, 32] was shown to have superb performance in measuring sequence dissimilarities. We compared the performances of RF with that based on the Manhattan distance and the $d^{*}_{2}$ statistic. The $d_{2}^{*}$ statistic between two sequences is defined as the uncentered correlation between two feature vectors according to the third definition of features.

We first calculated the average Manhattan distance between the frequency vectors of a viral sequence in the testing set with the viruses in the positive training data. We predicted a virus to infect the host if the distance is smaller than a given threshold. The predictions were then compared with the true infectious relationships to obtain the false positive rate and the true positive rate. By changing the threshold, we obtained the ROC curve and the AUC was calculated. The procedure was repeated 50 times in order to reduce the variation introduced by the selection of the negative testing data.

We did similar analysis using the $d_{2}^{*}$ dissimilarity measure. For $d_{2}^{*}$, the background Markov chain model was needed and we considered independent identically distributed (*i*.*i*.*d*.) model, first, second and third order Markov chains. We compared the performances based on Manhattan, $d_{2}^{*}$ under *i*.*i*.*d*., first, and second order MC background models and random forest with *k*=4, *k*=6 and *k*=8. The performances of different methods when *k*=6 are given in Table 5. The results based on *k*=4 and *k*=8 as well as the third order MC background model for $d_{2}^{*}$ are given in Additional file 1: Table S3.

**Table 5:** Comparison of AUC scores of RF (random forest) combined with word frequency vector with that based on Manhattan distance and $d_{2}^{*}$ statistic when *k*=6

	Manhattan	${d}_{2}^{*}$			RF-feat-1
		*i*.*i*.*d*.	1^*st*^−*m* *c*	2^*nd*^−*m* *c*	
Bacillus	0.829	0.752	0.873	0.851	0.863
Escherichia	0.880	0.833	0.958	0.945	0.856
Lactococcus	0.767	0.775	0.828	0.750	1.000
Mycobacterium	0.976	0.977	0.966	0.984	0.985
Pseudomonas	0.951	0.934	0.974	0.970	0.981
Salmonella	0.837	0.818	0.900	0.900	0.896
Staphylococcus	0.964	0.941	0.947	0.974	0.987
Synechococcus	0.929	0.906	0.994	0.993	0.978
Vibrio	0.841	0.733	0.854	0.817	0.940

For the background model of $d_{2}^{*}$ statistic, we considered independent identically distributed (*i*.*i*.*d*.) model, first and second order Markov chains

#### Identification of hosts of viral contigs in metagenomic studies

The primary motivation of our study is the identification of hosts of viral contigs in metagenomic studies. In viral metagenomic studies that viral DNA is separated from cellular DNA before sequencing, only viral genomes are sequenced (although there is usually some contaminating cellular DNA) and their host information is completely lost. It is important to match viral contigs with their corresponding hosts for the understanding of the virus-host infection dynamics in microbial communities. Because intact viral genomes are rarely recovered as contigs in viral metagenomic studies, the assembled viral contigs are generally much shorter than whole genome sequences and thus we study the performance of RF for the identification of bacterial hosts of viral contigs of different lengths. In order to achieve this objective, we studied the performance of RF for the prediction of hosts of viral contigs with different lengths: 1, 3, 5 kbps, and whole genome.

In addition to the RF method learned based on complete viral genomes, we can also learn the RF methods based on contigs of different lengths. We hypothesized that, to predict the hosts of contigs of a certain length, the best method should be learned from contigs with similar lengths. To test this hypothesis, we carried out the following study.

**Fig. 2:**
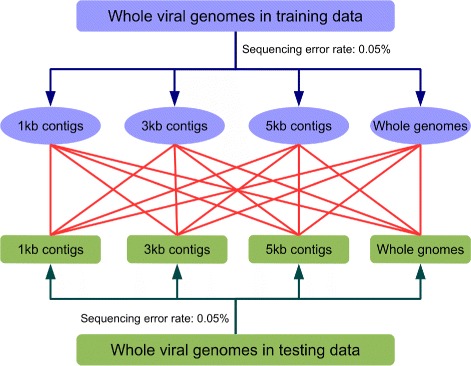
Scheme of the RF method for the prediction of hosts of viral contigs of different lengths. For each of the 9 main host genera, we produced 4 training datasets with differed sequence lengths by breaking the whole viral genomes into nonoverlapping contigs of lengths 1, 3, 5 kbps and the whole genomes with 0.05*%* sequencing errors added; Similarly, we also generated 4 different testing datasets with different contig lengths. We then evaluated the performances of RF for each training dataset with specific sequence length on all 4 testing datasets

For both training and testing data corresponding to the 9 bacterial host genera, we first broke the whole viral genomes into nonoverlapping contigs of lengths 1, 3, 5 kbps and whole genomes, respectively. To incorporate sequencing errors of NGS technologies, we modified the contigs with 0.05% sequencing error rate. When a sequencing error occurs at a nucleotide base, the original base was changed to any of the other three bases with equal probability. Figure 2 shows the schema of our study. We built RF predictors using the first feature of the training contigs using different lengths and predicted the hosts of the testing contigs. Table 6 shows the results. It can be seen from the table that the performances of the learned RF model based on contigs of 3 and 5 kbps are similar and are consistently among the best predictors for all the sequence contigs. When the contig length is 1 kbps, the word frequency vectors are not stable and have too much variation resulting in low performance of the learned model. On the other hand, the RF model based on the whole genome sequences does not perform well for short contigs of lengths 1 kbps or shorter. A potential explanation is that the frequency vectors of short contigs differ significantly from that of the whole genomes.

**Table 6:** The AUC scores of the RF method for the prediction of hosts of viral contigs with different lengths using the models built from contigs of different lengths

					Testing: 1 kb				
	Baci.	Esch.	Lact.	Myco.	Pseu.	Salm.	Stap.	Syne.	Virb.
Training: 1 kb	0.773	0.805	0.840	0.962	0.924	0.812	0.936	0.928	0.842
Training: 3 kb	0.819	0.857	0.833	0.977	0.959	0.818	0.955	0.960	0.858
Training: 5 kb	0.831	0.848	0.848	0.977	0.952	0.826	0.957	0.957	0.845
Training: wgs	0.821	0.718	0.818	0.886	0.833	0.792	0.948	0.890	0.774
					Testing: 3 kb				
	Baci.	Esch.	Lact.	Myco.	Pseu.	Salm.	Stap.	Syne.	Virb.
Training: 1 kb	0.766	0.862	0.842	0.979	0.947	0.843	0.961	0.961	0.880
Training: 3 kb	0.823	0.878	0.868	0.980	0.975	0.866	0.966	0.967	0.898
Training: 5 kb	0.850	0.899	0.889	0.985	0.978	0.880	0.976	0.983	0.917
Training: wgs	0.854	0.827	0.885	0.967	0.952	0.872	0.976	0.951	0.876
					Testing: 5 kb				
	Baci.	Esch.	Lact.	Myco.	Pseu.	Salm.	Stap.	Syne.	Virb.
Training: 1 kb	0.768	0.870	0.822	0.982	0.955	0.838	0.964	0.972	0.867
Training: 3 kb	0.827	0.900	0.869	0.985	0.977	0.871	0.974	0.986	0.904
Training: 5 kb	0.852	0.890	0.883	0.986	0.978	0.883	0.972	0.972	0.907
Training: wgs	0.858	0.865	0.888	0.983	0.970	0.882	0.979	0.965	0.900
					Testing: wgs				
	Baci.	Esch.	Lact.	Myco.	Pseu.	Salm.	Stap.	Syne.	Virb.
Training: 1 kb	0.778	0.860	0.814	0.984	0.960	0.812	0.971	0.981	0.896
Training: 3 kb	0.854	0.901	0.817	0.994	0.988	0.884	0.989	0.994	0.930
Training: 5 kb	0.870	0.923	0.861	0.994	0.992	0.889	0.992	0.996	0.934
Training: wgs	0.862	0.859	1.0	0.985	0.979	0.893	0.987	0.981	0.938

### Estimation of the reliability of observed virus-host infectious associations from viral-tagging experiments

The above studies showed that RF with the first feature performs well in predicting contigs coming from viral genomes infecting a host. For the host Synechococcus, the best word length is *k*=6. From the RF model that was trained by 30 positive viral genomes and 30 negative viral genomes, for any viral sequence to be predicted, a score between (0, 1) can be calculated. We first calculated the scores of the 17 positive viral genomes in the testing data, and we also calculated the scores of the negative viral genomes except the 30 negative genomes that were used in training the model. As stated in the “Methods” section, We assumed that the scores for the negative and positive sequences follow beta distributions and the corresponding parameters were estimated using moment estimators.

**Fig. 3:**
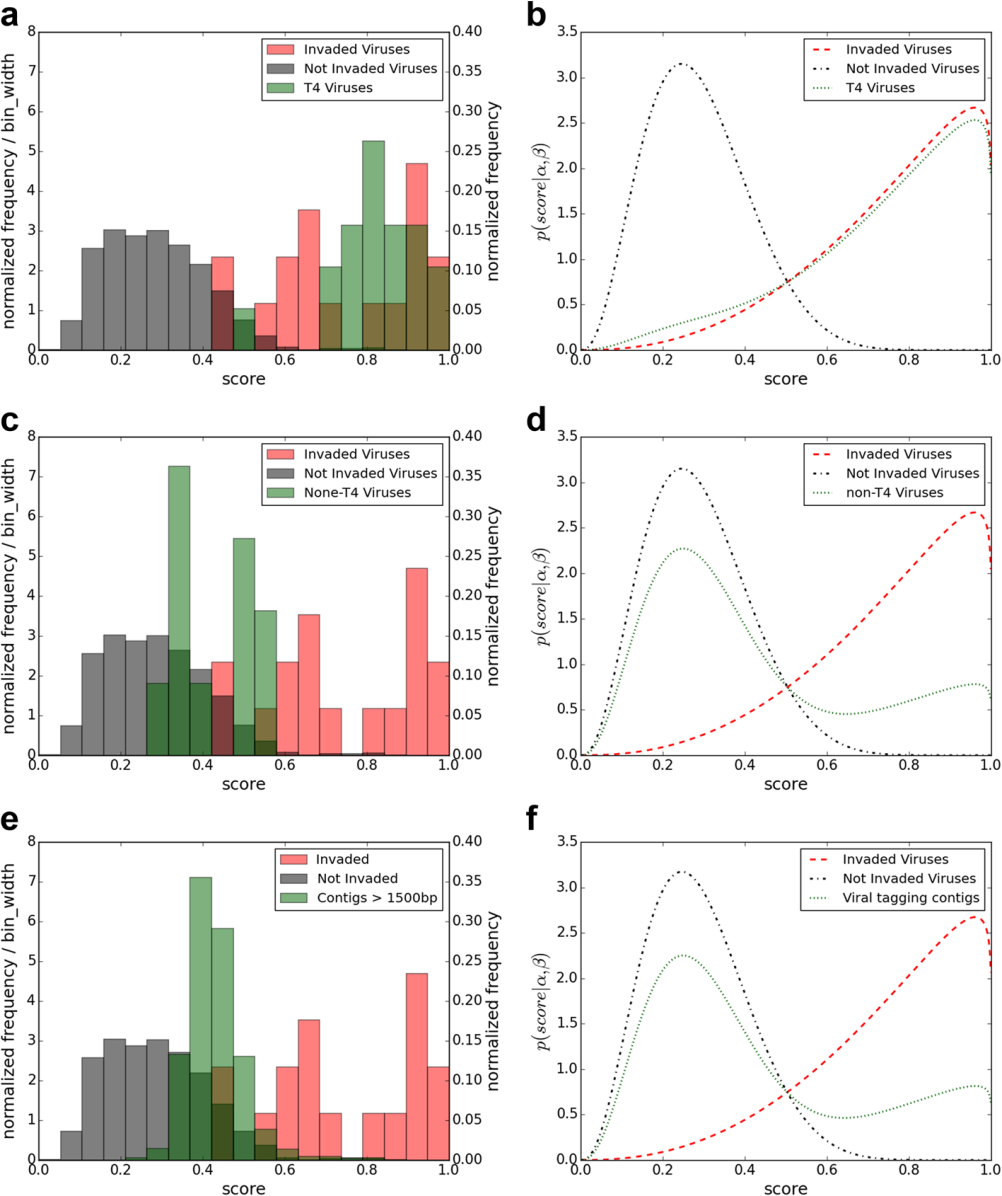
The histograms of the RF scores for the viral sequences in the negative and positive data sets, and **a** T4-like, **c** non-T4-like, and **e** viral contigs, respectively. The corresponding fitted density functions are given as **b**, **d**, and **f**, respectively. In all of the 6 subfigures, the horizontal axis is the prediction scores. In **a**, **c** and **e**, the *right y-axis* indicates the fraction and the *left y-axis* indicates the fraction divided by the bin-size

For the 30 candidate viral genomes identified to infect Synechococcus, we calculated the RF scores of the 19 T4-like viruses and 11 non-T4-like viruses. Figure 3 a and c shows the histograms of the RF scores of the viral sequences from the positive and negative testing datasets, and (a) the T4-like and (c) non-T4-like candidate genomes, respectively. The histogram of the scores of the T4-like viruses has a significant overlap with that for the positive testing data set. This observation strongly suggests that most of the identified T4-like viruses do infect Synechococcus. On the other hand, the histogram for the scores of the identified non-T4-like viruses peaked in the bins of viruses not infecting the host Synechococcus, but mixed to a small extent with the viruses that infect the host Synechococcus. This observation raises doubts that most of the identified non-T4-like viruses infect the host.

We then estimated the fraction of true infectious viruses using the maximum likelihood approach described in the “Methods” section. According to the distribution of positive testing viruses and negative viruses with respect to the host Synechococcus, the fitted distributions for the positive and negative viruses are *Φ*_1_(3.35,1.10) and *Φ*_0_(3.54,8.78), respectively. The maximum likelihood estimate of *γ* is 0.949 with the 95% confidence interval [ 0.933,0.964] for the T4-like viruses. The estimated *γ* is 0.288 with the 95% confidence interval [ 0.265,0.311]. The fitted density functions are given in Fig. 3 b and d, respectively.

We also used the same method to estimate the fraction of 1661 contigs with lengths at least 1.5 kbps from the viral tagging experiment with the host Synechococcus [26]. This fraction was estimated at 30.4% with 95% confidence interval [0.287, 0.320] (Fig. 3 e, f).

## Discussion and conclusions

In this paper, we developed methods to predict if a given viral DNA sequence (genome or large contig) comes from a virus that infects a particular host. First of all, we implemented five supervised learning methods including logistic regression, SVM with RBF kernel, random forest, Gaussian naive Bayesian and Bernoulli naive Bayesian with four features proposed based on word frequency with various orders of Markov chains as background models for viral sequences. We compared different machine learning methods and different feature representations based on nine host genera with at least 45 infectious associated viruses. We concluded that RF outperforms other methods with less dependence on sequence background model. For the four proposed feature representations, the relative word frequency representation (first feature) has the benefit of simplicity and has better or similar performances as other features. Besides, for word length selection, we compared the performance of RF using *k*=4, *k*=6 and *k*=8. For 6 out of 9 host genera, the performance of RF based on *k*=6 is the best. For host genera, Escherichia, Mycobacterium and Staphylococcus, *k*=4 performed slightly better than *k*=6. When choosing word length *k*=8, the performance of RF is lowered for all nine host genera.

Second, for all the nine main host genera, we studied the effect of contig length on the performance of RF for predicting the virus host infectious relationship. According to our simulation result, constructing the model by using contigs with lengths 3 or 5 kbps performs generally well for contigs with length from 1 kbps to the whole genome.

Third, we developed a maximum likelihood approach for estimating the fraction of viruses infecting a bacterial host in viral tagging expriment [26] based on word frequencies. We focused on two types of viruses: T4-like viruses and non-T4-like viruses. We showed that about 95% of the identified T4-like viruses appear to infect Synechococcus. On the other hand, only about 29% of the identified non-T4-like viruses and 30% of the contigs over 1.5 kbps have sequence word patterns that matched known Synechococcus viruses, and this raises doubts if the others actually infect Synechococcus. The scores for the contigs based on RF can also be used to prioritize the contigs for infection.

Finally, as viruses infecting their hosts can lead to changes in the metabolic rates, cell fates and functions of some of the host genes and therefore impact the whole community, it is significant to study virus-host infections. Our study not only has the potential in predicting future novel virus-host associations, but also can be applied to estimate the fraction of true infectious associations in high-throughput experiment such as viral tagging and SAG (single-cell amplified genomes).

Our study also has some limitations. First, the machine learning methods depend on relative large number of viruses infecting particular host genera. To meet this requirement, we studied only nine hosts. Many bacteria are available and most of them do not have viruses identified to infect them yet. Therefore, machine learning methods can not be applied to such potential hosts. Second, we only explored four feature representations of viruses based on word counts. Other viral sequence representations maybe more effective in the identification of viruses infecting particular hosts. For example, we can allow some mismatches or gaps for particular words for word counting [45, 46]. Finally, there are many variations for a particular machine leaning method and we only implemented one version. For example, we only used RBF kernel for SVM. Other kernels such as polynomial, exponential, and hyperbolic tangent kernels [25] may give better results. These are the topics for future studies. Despite these limitations, we clearly showed that RF can be used to predict viruses infecting particular hosts with very high accuracy.

## Additional file

Additional file 1 Supplementary_Material [47]. Detailed data description, supplementary methods description and implementation. (PDF 89 kb)
