# Supplementary material for: Prediction of virus-host infectious association by supervised learning methods
Source: BMC Bioinformatics. 2017 Mar 14;18(Suppl 3):60. doi: 10.1186/s12859-017-1473-7 (PMC5374558; doi:10.1186/s12859-017-1473-7)
Supplement: Additional file 1 — Supplementary_Material [47]. Detailed data description, supplementary methods description and implementation. (PDF 89 kb) [file 12859_2017_1473_MOESM1_ESM.pdf]

Prediction of virus-host infectious association by supervised learning methods

Mengge Zhang[??], Lianping Yang[??], Jie Ren[??], Nathan Ahlgren[??], Jed A. Fuhrman[??] and Fengzhu Sun[??,??*]

[*]Correspondence: fsun@usc.edu
[??]Molecular and Computational Biology Program, University of Southern California, Los Angeles, California, USA
Full list of author information is available at the end of the article

Additional file 1

Supplementary data description:

The NCBI phage genome database currently contains 1,426 completely sequenced viral genomes with precisely identified hosts. Among all the bacteria at the genus level, we focus on 9 bacterial genera with at least 45 identified infectious viuses. Table S1 shows the 9 bacterial host genera and the number of viruses identified to infect the genera until a given year from 2010 to 2015.

Table S1: The number of viruses identified to infect nine bacterial host genera up to the respective year.

Bacteria	Years					
	2010	2011	2012	2013	2014	2015
Bacillus	18	20	31	53	62	62
Escherichia	86	97	141	145	173	173
Lactococcus	48	48	48	49	55	55
Mycobacterium	34	38	89	172	215	218
Pseudomonas	34	39	57	68	96	96
Salmonella	16	22	32	47	52	54
Staphylococcus	21	24	43	52	62	63
Synechococcus	14	26	30	42	47	47
Vibrio	25	32	39	58	67	68

Supplementary methods:

AUC score:

AUC is the abbreviation for the “Area Under the Curve” for ROC (receiver operating characteristic) curves, which illustrates the performance of binary classifiers with varying discrimination threshold. The ROC curve is created by plotting the true positive rate (along the vertical axis) against the false positive rate (along the horizontal axis) with changing thresholds. Larger AUC score indicates better performance of the binary classifier.

Random forest

Decision tree

Decision Tree is a non-parametric supervised learning method commonly used for classification. From top to down, the decision tree has decision nodes and leaf nodes, where at each decision node there are two or more branches corresponding to different outcomes of decision, and each leaf node of the tree represents a final classification result of the data point. In our model, each decision node tests if the feature vector satisfies a certain threshold and makes a branching decision based on the outcome of the test, and the leaf node represents a classification label for the feature vector.

Random forest

Let $\{(\mathbf{x_i}, y_i), i = 1, 2, \cdots, n\}$ be the original training dataset, $\mathbf{x_i}$ represent the feature vector, and y_i represent the class label of the i-th object. The random forest method consists of the following steps:

1 Randomly select n samples from the original training data $\{(\mathbf{x_i}, y_i), i = 1, 2, \cdots, n\}$ with replacement;
2 Randomly select a subset of features in the feature vector and fit a decision tree based on the random selected samples in step-1 with the subset of features;
3 Repeat step-1 and step-2 for B times and denote $\hat{f}_b(x)$ $(1 \leq b \leq B)$ as the decision function of the decision tree trained from each random sampling;
4 Given the feature vector $\mathbf{x}$ of a test sample, the prediction will be the voting for the decisions from all B decision trees: $\hat{f}(x) = \sum_{b=1}^{B} \hat{f}_b(x)$.

The random sampling in step-1 is used to decrease the variance of the decision tree without adding bias to the model, and selecting a random subset of the features instead of using all of the features as in step-2 can de-correlate some features acting as strong predictors.

Naive Bayes

Denote Y as the binary random variable of the virus label and $\mathbf{x}$ as the feature vector, then by Bayes' theorem,

$$p(Y|\mathbf{x}) = \frac{p(Y)p(\mathbf{x}|Y)}{p(\mathbf{x})} \quad \Longrightarrow \quad \begin{cases} p(Y=1|\mathbf{x}) = \alpha \cdot p(Y=1)p(\mathbf{x}|Y=1) \\ p(Y=0|\mathbf{x}) = \alpha \cdot p(Y=0)p(\mathbf{x}|Y=1) \end{cases}$$

where $c = \frac{1}{p(\mathbf{x})}$. The prediction score is then given by

$$\hat{y} = \alpha \cdot p(Y=1)p(\mathbf{x}|Y=1) = 1 - \alpha \cdot p(Y=0)p(\mathbf{x}|Y=0)$$

If $\hat{y} > 0.5$, the virus is classified into the infectious class; Otherwise into the not infectious class. Depending on the different presumed distributions of $p(\mathbf{x}|Y)$, the naive Bayes approach can be subdevided into Gaussian naive Bayes and Bernoulli naive Bayes.

Gaussian naive Bayes

Let C_1 be the class of viruses with label $y = 1$ and C_0 be the class of viruses with label $y = 0$. Let μ_1 and $\mathbf{\Sigma}_1$ be the mean and covariance matrix for $\mathbf{x_i}$'s $\in C_1$. Similarly, let μ_0

and $\mathbf{\Sigma}_0$ be the mean and covariance matrix for $\mathbf{x_j}$'s $\in C_0$. Then for any feature vector $\mathbf{x}$ from testing data:

$$p(\mathbf{x}=\mathbf{v}|C_1) = \frac{1}{\sqrt{(2\pi)^k|\mathbf{\Sigma}_1|}} \exp\left(-\frac{1}{2}(\mathbf{x}-\mu_1)^T\mathbf{\Sigma}_1^{-1}(\mathbf{x}-\mu_1)\right)$$

$$p(\mathbf{x}=\mathbf{v}|C_0) = \frac{1}{\sqrt{(2\pi)^k|\mathbf{\Sigma}_0|}} \exp\left(-\frac{1}{2}(\mathbf{x}-\mu_0)^T\mathbf{\Sigma}_0^{-1}(\mathbf{x}-\mu_0)\right)$$

Bernoulli naive Bayes
Let $\mathbf{x}_i = (x_i^1, x_i^2, \cdots, x_i^K)$, where K is the dimension of the feature vectors. Similar as in Gaussian naive Bayes, let C_1 be class that the labels of data in it are $y=1$, C_0 be class that the labels of data in it are $y=0$. Then for $\mathbf{x_i}$'s $\in C_1$, we can calculate $p_{1k} = \frac{\sum_{i=1}^{N_1} sgn(x_i^k)}{N_1}$, where N_1 is the size of class C_1 and $k \in \{1,\cdots,K\}$. For $\mathbf{x_j}$'s $\in C_0$, $p_{0k} = \frac{\sum_{i=1}^{N_0} sgn(x_i^k)}{N_0}$, where N_0 is the size of class C_0. Then for any feature vector $\mathbf{x}$ from the testing data:

$$p(\mathbf{x}|C_1) = \prod_{k=1}^{K} p_{1k}^{sgn(x_k)}(1-p_{1k})^{1-sgn(x_k)}$$

$$p(\mathbf{x}|C_0) = \prod_{k=1}^{K} p_{0k}^{sgn(x_k)}(1-p_{0k})^{1-sgn(x_k)}$$

*Note: $sgn(x)$ is the sign function: $sgn(x) = \begin{cases} 1 & \text{if } x > 0 \\ 0 & \text{if } x = 0 \end{cases}$, in our case, the entries of the feature vectors are all non-negative.

Implementation

When comparing feature-method combinations, for all 9 different host, negative training data and negative testing data were chosen randomly for 50 times. The positive training data and positive testing data are the same for the 50 runs of the pipeline. For each of the 50 datasets, we trained the model purely based on the training data, and then test the performance of the method on the testing data.

When apply our method on the T4-like / non-T4-like viruses with host Synechococcus, we also repeated our procedure for 50 times with different negative training data. The confidence intervals were calculated based on the results of from the 50 runs of the procedure.

The construction of the feature vectors with different word lengths and different sequence background models were implemented with c++. The supervised learning methods parts were implemented with python package scikit-learn.

Additional Supplementary Tables

Table S3: AUC scores of unsupervised learning based on the average Manhattan distance and d_2^* dissimilarity of viruses in the testing data with the viruses in the positive training data. Three different word lengths : $k = 4$, $k = 6$, and $k = 8$; And four different background models were applied for d_2^* dissimilarity.

$\mathbf{k = 4}$		$\mathbf{d_2^*}$			
	Manhattan	$i.i.d.$	$1^{st} - mc$	$2^{nd} - mc$	$3^{rd} - mc$
Bacillus	0.826	0.741	0.867	0.778	0.780
Escherichia	0.828	0.772	0.913	0.860	0.602
Lactococcus	0.844	0.778	0.697	0.533	0.794
Mycobacterium	0.977	0.975	0.938	0.962	0.856
Pseudomonas	0.941	0.921	0.941	0.910	0.936
Salmonella	0.818	0.762	0.894	0.868	0.818
Staphylococcus	0.954	0.930	0.861	0.945	0.896
Synechococcus	0.919	0.890	0.940	0.946	0.696
Vibrio	0.852	0.715	0.807	0.754	0.696
$\mathbf{k = 6}$		$\mathbf{d_2^*}$			
	Manhattan	$i.i.d.$	$1^{st} - mc$	$2^{nd} - mc$	$3^{rd} - mc$
Bacillus	0.829	0.752	0.873	0.851	0.904
Escherichia	0.880	0.833	0.958	0.945	0.939
Lactococcus	0.767	0.775	0.828	0.750	0.836
Mycobacterium	0.976	0.977	0.966	0.984	0.985
Pseudomonas	0.951	0.934	0.974	0.970	0.990
Salmonella	0.837	0.818	0.900	0.900	0.828
Staphylococcus	0.964	0.941	0.947	0.974	0.983
Synechococcus	0.929	0.906	0.994	0.993	0.991
Vibrio	0.841	0.733	0.854	0.817	0.853
$\mathbf{k = 8}$		$\mathbf{d_2^*}$			
	Manhattan	$i.i.d.$	$1^{st} - mc$	$2^{nd} - mc$	$3^{rd} - mc$
Bacillus	0.812	0.774	0.858	0.848	0.873
Escherichia	0.873	0.838	0.957	0.936	0.945
Lactococcus	0.861	0.761	0.872	0.828	0.814
Mycobacterium	0.986	0.982	0.984	0.983	0.992
Pseudomonas	0.961	0.938	0.981	0.982	0.989
Salmonella	0.770	0.822	0.887	0.904	0.850
Staphylococcus	0.946	0.928	0.963	0.967	0.977
Synechococcus	0.896	0.881	0.972	0.975	0.984
Vibrio	0.738	0.694	0.803	0.792	0.825

Table S2

Table S2:The average AUC scores for all the feature-method combinations. Three different word length (4, 6, 8) and four different background models (i.i.d. model, 1st, 2nd and 3rd order Markov chains) were used. For each feature-method combination trained purely based on the training data, we apply it onto the testing data and calculate the AUC score. We repeated our pipeline for 50 times with different negative training data and negative testing data each time, and then we calculated the average AUC score.

Host: **Bacillus**		i.i.d. model			1st order mc		
		$k=4$	$k=6$	$k=8$	$k=4$	$k=6$	$k=8$
Feature-1	Logistic Regression	0.849	0.854	0.793	0.849	0.854	0.793
	SVM	0.744	0.750	0.700	0.744	0.750	0.700
	Random forest	0.856	0.864	0.823	0.856	0.864	0.823
	Gaussian NB	0.811	0.770	0.731	0.811	0.770	0.731
	Bernoulli NB	0.689	0.787	0.782	0.689	0.787	0.782
Feature-2	Logistic Regression	0.831	0.832	0.822	0.850	0.847	0.808
	SVM	0.777	0.837	0.723	0.798	0.823	0.671
	Random forest	0.855	0.862	0.821	0.872	0.881	0.850
	Gaussian NB	0.811	0.723	0.500	0.768	0.766	0.498
	Bernoulli NB	0.784	0.771	0.707	0.803	0.808	0.663
Feature-3	Logistic Regression	0.833	0.856	0.833	0.823	0.844	0.818
	SVM	0.547	0.563	0.703	0.563	0.570	0.615
	Random forest	0.843	0.861	0.805	0.866	0.874	0.807
	Gaussian NB	0.758	0.740	0.713	0.740	0.776	0.640
	Bernoulli NB	0.784	0.771	0.707	0.803	0.808	0.663
Feature-4	Logistic Regression	0.783	0.824	0.820	0.845	0.852	0.837
	SVM	0.823	0.845	0.790	0.786	0.766	0.618
	Random forest	0.852	0.863	0.803	0.886	0.879	0.834
	Gaussian NB	0.757	0.700	0.627	0.769	0.690	0.613
	Bernoulli NB	0.784	0.771	0.707	0.803	0.808	0.663
		2nd order mc			3rd order mc		
		$k=4$	$k=6$	$k=8$	$k=4$	$k=6$	$k=8$
Feature-1	Logistic Regression	0.849	0.855	0.793	0.849	0.855	0.793
	SVM	0.744	0.750	0.700	0.744	0.750	0.700
	Random forest	0.856	0.864	0.823	0.856	0.864	0.823
	Gaussian NB	0.811	0.770	0.731	0.811	0.770	0.731
	Bernoulli NB	0.689	0.787	0.782	0.689	0.787	0.782
Feature-2	Logistic Regression	0.854	0.855	0.821	0.736	0.766	0.736
	SVM	0.618	0.842	0.623	0.537	0.595	0.521
	Random forest	0.901	0.893	0.840	0.864	0.843	0.835
	Gaussian NB	0.798	0.803	0.492	0.505	0.698	0.490
	Bernoulli NB	0.774	0.768	0.689	0.644	0.698	0.673
Feature-3	Logistic Regression	0.835	0.865	0.826	0.745	0.836	0.810
	SVM	0.595	0.574	0.560	0.565	0.640	0.547
	Random forest	0.878	0.871	0.802	0.903	0.861	0.807
	Gaussian NB	0.748	0.755	0.590	0.497	0.665	0.536
	Bernoulli NB	0.774	0.768	0.689	0.644	0.698	0.673
Feature-4	Logistic Regression	0.850	0.860	0.837	0.783	0.841	0.823
	SVM	0.708	0.742	0.557	0.755	0.673	0.511
	Random forest	0.872	0.859	0.821	0.912	0.851	0.816
	Gaussian NB	0.690	0.668	0.605	0.498	0.660	0.584
	Bernoulli NB	0.774	0.768	0.689	0.644	0.695	0.673

Host: **Escherichia**		i.i.d. model			1st order mc		
		$k=4$	$k=6$	$k=8$	$k=4$	$k=6$	$k=8$
Feature-1	Logistic Regression	0.882	0.909	0.915	0.882	0.909	0.915
	SVM	0.652	0.602	0.500	0.652	0.602	0.500
	Random forest	0.878	0.858	0.807	0.878	0.858	0.807
	Gaussian NB	0.780	0.825	0.491	0.780	0.825	0.491
	Bernoulli NB	0.602	0.463	0.395	0.602	0.463	0.395
Feature-2	Logistic Regression	0.777	0.793	0.813	0.809	0.800	0.769
	SVM	0.861	0.911	0.800	0.780	0.913	0.809
	Random forest	0.882	0.857	0.807	0.900	0.845	0.776
	Gaussian NB	0.780	0.808	0.525	0.755	0.514	0.559
	Bernoulli NB	0.705	0.714	0.619	0.844	0.725	0.544
Feature-3	Logistic Regression	0.781	0.778	0.748	0.779	0.794	0.726
	SVM	0.789	0.755	0.775	0.773	0.769	0.797
	Random forest	0.877	0.859	0.809	0.885	0.856	0.8081
	Gaussian NB	0.592	0.605	0.752	0.617	0.753	0.639
	Bernoulli NB	0.705	0.714	0.619	0.844	0.725	0.544
Feature-4	Logistic Regression	0.778	0.794	0.778	0.818	0.839	0.815
	SVM	0.910	0.902	0.850	0.858	0.708	0.500
	Random forest	0.874	0.863	0.826	0.904	0.860	0.830
	Gaussian NB	0.778	0.817	0.777	0.858	0.869	0.703
	Bernoulli NB	0.705	0.714	0.619	0.844	0.725	0.544
		2nd order mc			3rd order mc		
		$k=4$	$k=6$	$k=8$	$k=4$	$k=6$	$k=8$
Feature-1	Logistic Regression	0.882	0.909	0.915	0.882	0.909	0.915
	SVM	0.652	0.602	0.500	0.652	0.602	0.500
	Random forest	0.878	0.858	0.807	0.878	0.858	0.807
	Gaussian NB	0.780	0.825	0.491	0.780	0.825	0.491
	Bernoulli NB	0.602	0.463	0.395	0.602	0.463	0.395
Feature-2	Logistic Regression	0.789	0.832	0.780	0.747	0.762	0.700
	SVM	0.608	0.914	0.775	0.484	0.861	0.728
	Random forest	0.848	0.795	0.758	0.839	0.754	0.754
	Gaussian NB	0.797	0.544	0.744	0.452	0.467	0.725
	Bernoulli NB	0.633	0.584	0.458	0.606	0.592	0.455
Feature-3	Logistic Regression	0.747	0.788	0.711	0.791	0.764	0.693
	SVM	0.811	0.799	0.795	0.464	0.827	0.753
	Random forest	0.840	0.803	0.775	0.864	0.753	0.772
	Gaussian NB	0.642	0.703	0.573	0.461	0.717	0.573
	Bernoulli NB	0.633	0.584	0.458	0.606	0.592	0.455
Feature-4	Logistic Regression	0.776	0.830	0.799	0.766	0.824	0.776
	SVM	0.627	0.548	0.500	0.686	0.500	0.500
	Random forest	0.837	0.815	0.821	0.923	0.790	0.816
	Gaussian NB	0.717	0.808	0.678	0.483	0.795	0.667
	Bernoulli NB	0.633	0.584	0.458	0.603	0.588	0.455

Host: **Lactococcus**		i.i.d. model			1st order mc		
		$k=4$	$k=6$	$k=8$	$k=4$	$k=6$	$k=8$
Feature-1	Logistic Regression	0.922	0.950	0.881	0.922	0.950	0.881
	SVM	0.692	0.692	0.742	0.692	0.692	0.742
	Random forest	0.972	1.00	0.988	0.972	1.00	0.988
	Gaussian NB	0.642	0.825	0.692	0.642	0.825	0.692
	Bernoulli NB	0.525	0.767	0.633	0.525	0.767	0.633
Feature-2	Logistic Regression	0.957	0.968	0.950	0.945	0.951	0.891
	SVM	0.883	0.808	0.583	0.583	0.667	0.500
	Random forest	0.975	1.000	0.981	1.000	0.928	0.933
	Gaussian NB	0.642	0.717	0.500	0.583	0.583	0.500
	Bernoulli NB	0.792	0.767	0.658	0.583	0.500	0.583
Feature-3	Logistic Regression	0.943	0.952	0.950	0.938	0.954	0.943
	SVM	0.500	0.500	0.500	0.500	0.500	0.500
	Random forest	1.000	0.938	0.942	0.974	0.900	0.967
	Gaussian NB	0.492	0.467	0.500	0.500	0.500	0.500
	Bernoulli NB	0.792	0.767	0.658	0.583	0.500	0.583
Feature-4	Logistic Regression	0.944	0.950	0.950	0.915	0.947	0.943
	SVM	0.717	0.775	0.583	0.575	0.583	0.500
	Random forest	0.978	0.969	0.908	0.989	0.972	0.969
	Gaussian NB	0.875	0.825	0.500	0.500	0.608	0.500
	Bernoulli NB	0.792	0.767	0.658	0.583	0.500	0.583

		2nd order mc			3rd order mc		
		$k=4$	$k=6$	$k=8$	$k=4$	$k=6$	$k=8$
Feature-1	Logistic Regression	0.922	0.950	0.881	0.922	0.950	0.881
	SVM	0.692	0.692	0.742	0.692	0.692	0.742
	Random forest	0.972	1.00	0.988	0.972	1.00	0.988
	Gaussian NB	0.642	0.825	0.692	0.642	0.825	0.692
	Bernoulli NB	0.525	0.767	0.633	0.525	0.767	0.633
Feature-2	Logistic Regression	0.955	0.937	0.863	0.947	0.888	0.808
	SVM	0.667	0.583	0.500	0.550	0.558	0.500
	Random forest	0.942	0.914	0.936	0.994	0.897	0.894
	Gaussian NB	0.583	0.608	0.500	0.775	0.642	0.500
	Bernoulli NB	0.583	0.500	0.583	1.000	0.500	0.583
Feature-3	Logistic Regression	0.950	0.967	0.954	0.947	0.931	0.963
	SVM	0.500	0.500	0.500	0.575	0.583	0.500
	Random forest	0.906	0.913	0.967	1.000	0.911	0.967
	Gaussian NB	0.467	0.500	0.500	0.750	0.500	0.500
	Bernoulli NB	0.583	0.500	0.583	1.000	0.500	0.583
Feature-4	Logistic Regression	0.947	0.947	0.943	0.936	0.943	0.943
	SVM	0.650	0.525	0.500	0.600	0.500	0.500
	Random forest	0.903	0.938	0.986	1.000	0.917	0.979
	Gaussian NB	0.583	0.608	0.500	0.667	0.658	0.500
	Bernoulli NB	0.583	0.500	0.583	1.000	0.500	0.583

Host: **Mycobacterium**		i.i.d. model $k=4$	i.i.d. model $k=6$	i.i.d. model $k=8$	1st order mc $k=4$	1st order mc $k=6$	1st order mc $k=8$
Feature-1	Logistic Regression	0.942	0.947	0.944	0.942	0.947	0.944
	SVM	0.802	0.757	0.715	0.802	0.757	0.715
	Random forest	0.987	0.985	0.984	0.987	0.985	0.984
	Gaussian NB	0.937	0.933	0.804	0.937	0.933	0.804
	Bernoulli NB	0.613	0.920	0.926	0.613	0.920	0.926
Feature-2	Logistic Regression	0.928	0.939	0.926	0.928	0.930 5	0.896
	SVM	0.941	0.980	0.924	0.939	0.980	0.774
	Random forest	0.988	0.985	0.983	0.982	0.978	0.984
	Gaussian NB	0.937	0.946	0.680	0.935	0.941	0.683
	Bernoulli NB	0.920	0.911	0.920	0.920	0.937	0.924
Feature-3	Logistic Regression	0.928	0.938	0.921	0.930	0.929	0.903
	SVM	0.846	0.870	0.946	0.872	0.894	0.961
	Random forest	0.993	0.992	0.989	0.986	0.983	0.981
	Gaussian NB	0.952	0.950	0.946	0.935	0.959	0.859
	Bernoulli NB	0.920	0.911	0.920	0.920	0.937	0.924
Feature-4	Logistic Regression	0.919	0.939	0.925	0.937	0.942	0.933
	SVM	0.961	0.989	0.983	0.891	0.904	0.946
	Random forest	0.992	0.989	0.986	0.986	0.985	0.982
	Gaussian NB	0.944	0.939	0.939	0.944	0.944	0.941
	Bernoulli NB	0.920	0.911	0.920	0.920	0.937	0.924
		2nd order mc $k=4$	2nd order mc $k=6$	2nd order mc $k=8$	3rd order mc $k=4$	3rd order mc $k=6$	3rd order mc $k=8$
Feature-1	Logistic Regression	0.942	0.947	0.944	0.942	0.947	0.944
	SVM	0.802	0.757	0.715	0.802	0.757	0.715
	Random forest	0.987	0.985	0.984	0.987	0.985	0.984
	Gaussian NB	0.937	0.933	0.804	0.937	0.933	0.804
	Bernoulli NB	0.613	0.920	0.926	0.613	0.920	0.926
Feature-2	Logistic Regression	0.897	0.921	0.728	0.919	0.900	0.843
	SVM	0.915	0.907	0.654	0.530	0.944	0.628
	Random forest	0.982	0.978	0.984	0.985	0.975	0.984
	Gaussian NB	0.944	0.944	0.683	0.715	0.839	0.704
	Bernoulli NB	0.950	0.952	0.922	0.874	0.950	0.920
Feature-3	Logistic Regression	0.924	0.925 5	0.895	0.910	0.908	0.876
	SVM	0.922	0.928	0.937	0.746	0.967	0.952
	Random forest	0.985	0.984	0.983	0.986	0.983	0.981
	Gaussian NB	0.957	0.963	0.759	0.689	0.948	0.757
	Bernoulli NB	0.950	0.952	0.922	0.874	0.950	0.920
Feature-4	Logistic Regression	0.918	0.940	0.931	0.912	0.937	0.929
	SVM	0.935	0.928	0.952	0.800	0.924	0.941
	Random forest	0.988	0.984	0.983	0.965	0.982	0.982
	Gaussian NB	0.948	0.950	0.946	0.694	0.950	0.946
	Bernoulli NB	0.950	0.952	0.922	0.874	0.952	0.917

Host: **Pseudomonas**		i.i.d. model			1st order mc		
		$k=4$	$k=6$	$k=8$	$k=4$	$k=6$	$k=8$
Feature-1	Logistic Regression	0.938	0.947	0.943	0.937	0.947	0.943
	SVM	0.673	0.695	0.738	0.673	0.695	0.738
	Random forest	0.978	0.981	0.967	0.978	0.981	0.967
	Gaussian NB	0.838	0.854	0.857	0.838	0.854	0.857
	Bernoulli NB	0.595	0.789	0.861	0.595	0.789	0.861
Feature-2	Logistic Regression	0.923	0.927	0.911	0.910	0.905	0.887
	SVM	0.911	0.959	0.946	0.873	0.963	0.846
	Random forest	0.977	0.980	0.968	0.986	0.979	0.961
	Gaussian NB	0.838	0.857	0.863	0.923	0.938	0.882
	Bernoulli NB	0.848	0.845	0.861	0.941	0.934	0.893
Feature-3	Logistic Regression	0.918	0.925	0.905	0.921	0.914	0.892
	SVM	0.889	0.905	0.921	0.902	0.921	0.925
	Random forest	0.974	0.980	0.959	0.983	0.978	0.971
	Gaussian NB	0.805	0.852	0.875	0.934	0.946	0.921
	Bernoulli NB	0.848	0.845	0.861	0.941	0.934	0.893
Feature-4	Logistic Regression	0.917	0.929	0.906	0.935	0.933	0.917
	SVM	0.950	0.964	0.941	0.893	0.918	0.938
	Random forest	0.985	0.981	0.969	0.988	0.981	0.974
	Gaussian NB	0.854	0.850	0.873	0.938	0.896	0.886
	Bernoulli NB	0.848	0.845	0.861	0.941	0.934	0.893
		2nd order mc			3rd order mc		
		$k=4$	$k=6$	$k=8$	$k=4$	$k=6$	$k=8$
Feature-1	Logistic Regression	0.938	0.947	0.943	0.937	0.947	0.943
	GBR	0.946	0.908	0.891	0.946	0.908	0.891
	SVM	0.673	0.695	0.738	0.673	0.695	0.738
	Random forest	0.978	0.981	0.967	0.978	0.981	0.967
	Gaussian NB	0.838	0.854	0.857	0.838	0.854	0.857
	Bernoulli NB	0.595	0.789	0.861	0.595	0.789	0.861
Feature-2	Logistic Regression	0.898	0.895	0.873	0.851	0.866	0.864
	SVM	0.675	0.921	0.768	0.505	0.941	0.750
	Random forest	0.970	0.965	0.963	0.950	0.959	0.958
	Gaussian NB	0.929	0.886	0.861	0.586	0.877	0.868
	Bernoulli NB	0.893	0.896	0.891	0.809	0.929	0.888
Feature-3	Logistic Regression	0.895	0.909	0.890	0.801	0.890	0.875
	SVM	0.911	0.938	0.904	0.575	0.923	0.914
	Random forest	0.964	0.964	0.965	0.937	0.962	0.964
	Gaussian NB	0.934	0.955	0.889	0.552	0.975	0.879
	Bernoulli NB	0.893	0.896	0.891	0.809	0.929	0.888
Feature-4	Logistic Regression	0.917	0.921	0.905	0.747	0.917	0.903
	SVM	0.827	0.909	0.934	0.689	0.932	0.929
	Random forest	0.971	0.969	0.967	0.936	0.966	0.966
	Gaussian NB	0.886	0.893	0.882	0.557	0.895	0.882
	Bernoulli NB	0.893	0.896	0.891	0.796	0.929	0.888

Host: **Salmonella**		i.i.d. model			1st order mc		
		$k=4$	$k=6$	$k=8$	$k=4$	$k=6$	$k=8$
Feature-1	Logistic Regression	0.845	0.862	0.829	0.846	0.861	0.829
	SVM	0.600	0.584	0.557	0.600	0.584	0.557
	Random forest	0.889	0.896	0.891	0.889	0.896	0.891
	Gaussian NB	0.757	0.798	0.689	0.757	0.798	0.689
	Bernoulli NB	0.568	0.711	0.752	0.568	0.711	0.752
Feature-2	Logistic Regression	0.808	0.858	0.845	0.848	0.857	0.871
	SVM	0.743	0.784	0.711	0.832	0.864	0.741
	Random forest	0.889	0.894	0.891	0.932	0.915	0.912
	Gaussian NB	0.757	0.789	0.575	0.818	0.816	0.568
	Bernoulli NB	0.784	0.784	0.777	0.850	0.848	0.839
Feature-3	Logistic Regression	0.783	0.849	0.852	0.827	0.861	0.864
	SVM	0.727	0.773	0.805	0.766	0.782	0.780
	Random forest	0.890	0.895	0.880	0.937	0.918	0.897
	Gaussian NB	0.755	0.773	0.800	0.800	0.832	0.764
	Bernoulli NB	0.784	0.784	0.777	0.850	0.848	0.839
Feature-4	Logistic Regression	0.798	0.831	0.834	0.859	0.868	0.876
	SVM	0.814	0.818	0.821	0.859	0.850	0.602
	Random forest	0.908	0.895	0.897	0.933	0.921	0.904
	Gaussian NB	0.807	0.807	0.839	0.811	0.827	0.821
	Bernoulli NB	0.784	0.784	0.777	0.850	0.848	0.839
		2nd order mc			3rd order mc		
		$k=4$	$k=6$	$k=8$	$k=4$	$k=6$	$k=8$
Feature-1	Logistic Regression	0.845	0.862	0.829	0.846	0.861	0.829
	SVM	0.600	0.584	0.557	0.600	0.584	0.557
	Random forest	0.889	0.896	0.891	0.889	0.896	0.891
	Gaussian NB	0.757	0.798	0.689	0.757	0.798	0.689
	Bernoulli NB	0.568	0.711	0.752	0.568	0.711	0.752
Feature-2	Logistic Regression	0.845	0.864	0.863	0.637	0.815	0.819
	SVM	0.632	0.809	0.757	0.525	0.766	0.639
	Random forest	0.899	0.900	0.904	0.864	0.888	0.900
	Gaussian NB	0.827	0.780	0.568	0.584	0.777	0.573
	Bernoulli NB	0.791	0.809	0.777	0.652	0.736	0.768
Feature-3	Logistic Regression	0.817	0.861	0.850	0.623	0.837	0.827
	SVM	0.782	0.768	0.746	0.511	0.777	0.716
	Random forest	0.897	0.905	0.889	0.876	0.880	0.893
	Gaussian NB	0.807	0.800	0.741	0.552	0.709	0.698
	Bernoulli NB	0.791	0.809	0.777	0.652	0.736	0.768
Feature-4	Logistic Regression	0.841	0.865	0.857	0.626	0.848	0.842
	SVM	0.843	0.775	0.582	0.643	0.634	0.571
	Random forest	0.897	0.897	0.890	0.903	0.894	0.886
	Gaussian NB	0.807	0.809	0.768	0.575	0.809	0.741
	Bernoulli NB	0.791	0.809	0.777	0.664	0.736	0.768

Host: **Staphylococcus**		i.i.d. model			1st order mc		
		$k=4$	$k=6$	$k=8$	$k=4$	$k=6$	$k=8$
Feature-1	Logistic Regression	0.940	0.941	0.927	0.940	0.941	0.927
	SVM	0.740	0.788	0.748	0.740	0.788	0.748
	Random forest	0.993	0.987	0.983	0.993	0.987	0.983
	Gaussian NB	0.885	0.900	0.780	0.885	0.900	0.780
	Bernoulli NB	0.668	0.880	0.923	0.668	0.880	0.923
Feature-2	Logistic Regression	0.921	0.936	0.918	0.921	0.912	0.895
	SVM	0.913	0.968	0.885	0.890	0.963	0.705
	Random forest	0.993	0.987	0.983	0.985	0.982	0.975
	Gaussian NB	0.885	0.883	0.525	0.953	0.958	0.528
	Bernoulli NB	0.833	0.825	0.850	0.950	0.930	0.900
Feature-3	Logistic Regression	0.898	0.932	0.923	0.899	0.922	0.916
	SVM	0.850	0.875	0.868	0.875	0.880	0.905
	Random forest	0.990	0.984	0.981	0.984	0.980	0.981
	Gaussian NB	0.925	0.925	0.948	0.928	0.953	0.550
	Bernoulli NB	0.833	0.825	0.850	0.950	0.930	0.900
Feature-4	Logistic Regression	0.904	0.932	0.918	0.932	0.933	0.919
	SVM	0.878	0.893	0.935	0.808	0.913	0.913
	Random forest	0.991	0.987	0.985	0.990	0.9832	0.9894
	Gaussian NB	0.898	0.910	0.893	0.933	0.940	0.850
	Bernoulli NB	0.833	0.825	0.850	0.950	0.930	0.900
		2nd order mc			3rd order mc		
		$k=4$	$k=6$	$k=8$	$k=4$	$k=6$	$k=8$
Feature-1	Logistic Regression	0.940	0.941	0.927	0.940	0.941	0.927
	SVM	0.740	0.788	0.748	0.740	0.788	0.748
	Random forest	0.993	0.987	0.982	0.993	0.987	0.983
	Gaussian NB	0.885	0.900	0.780	0.885	0.900	0.780
	Bernoulli NB	0.668	0.880	0.923	0.668	0.880	0.923
Feature-2	Logistic Regression	0.905	0.909	0.885	0.910	0.861	0.846
	SVM	0.768	0.930	0.543	0.520	0.840	0.525
	Random forest	0.984	0.978	0.977	0.995	0.973	0.974
	Gaussian NB	0.953	0.923	0.528	0.763	0.818	0.525
	Bernoulli NB	0.958	0.920	0.900	0.960	0.918	0.905
Feature-3	Logistic Regression	0.909	0.924	0.912	0.928	0.917	0.908
	SVM	0.908	0.888	0.845	0.593	0.925	0.845
	Random forest	0.981	0.974	0.9807	0.993	0.970	0.977
	Gaussian NB	0.950	0.928	0.525	0.753	0.863	0.538
	Bernoulli NB	0.958	0.930	0.900	0.960	0.918	0.905
Feature-4	Logistic Regression	0.929	0.935	0.921	0.933	0.93	0.917
	SVM	0.868	0.908	0.888	0.858	0.938	0.828
	Random forest	0.986	0.982	0.986	0.989	0.979	0.979
	Gaussian NB	0.958	0.928	0.765	0.740	0.953	0.813
	Bernoulli NB	0.958	0.920	0.900	0.960	0.918	0.905

Host: **Synechococcus**		i.i.d. model			1st order mc		
		$k=4$	$k=6$	$k=8$	$k=4$	$k=6$	$k=8$
Feature-1	Logistic Regression	0.925	0.940	0.942	0.925	0.940	0.942
	SVM	0.706	0.735	0.500	0.706	0.735	0.500
	Random forest	0.965	0.978	0.955	0.965	0.980	0.954
	Gaussian NB	0.879	0.879	0.632	0.879	0.879	0.632
	Bernoulli NB	0.659	0.674	0.694	0.659	0.674	0.694
Feature-2	Logistic Regression	0.913	0.926	0.925	0.885	0.929	0.927
	SVM	0.921	0.929	0.747	0.794	0.800	0.665
	Random forest	0.965	0.978	0.957	0.956	0.967	0.925
	Gaussian NB	0.879	0.885	0.759	0.879	0.835	0.679
	Bernoulli NB	0.747	0.777	0.811	0.891	0.894	0.800
Feature-3	Logistic Regression	0.909	0.926	0.920	0.882	0.921	0.927
	SVM	0.529	0.647	0.718	0.582	0.715	0.753
	Random forest	0.935	0.960	0.897	0.917	0.923	0.864
	Gaussian NB	0.753	0.750	0.750	0.741	0.756	0.762
	Bernoulli NB	0.747	0.777	0.812	0.891	0.894	0.800
Feature-4	Logistic Regression	0.900	0.917	0.921	0.922	0.936	0.930
	SVM	0.888	0.927	0.768	0.888	0.903	0.741
	Random forest	0.960	0.981	0.947	0.978	0.981	0.974
	Gaussian NB	0.838	0.871	0.768	0.859	0.832	0.788
	Bernoulli NB	0.7471	0.777	0.812	0.891	0.894	0.800
		2nd order mc			3rd order mc		
		$k=4$	$k=6$	$k=8$	$k=4$	$k=6$	$k=8$
Feature-1	Logistic Regression	0.925	0.940	0.942	0.925	0.940	0.942
	SVM	0.706	0.735	0.500	0.706	0.735	0.500
	Random forest	0.966	0.977	0.960	0.966	0.977	0.960
	Gaussian NB	0.879	0.879	0.632	0.879	0.879	0.632
	Bernoulli NB	0.659	0.674	0.694	0.659	0.674	0.694
Feature-2	Logistic Regression	0.825	0.917	0.917	0.869	0.914	0.905
	SVM	0.729	0.803	0.662	0.562	0.853	0.585
	Random forest	0.915	0.966	0.916	0.941	0.969	0.901
	Gaussian NB	0.832	0.788	0.679	0.709	0.729	0.653
	Bernoulli NB	0.844	0.768	0.765	0.915	0.824	0.788
Feature-3	Logistic Regression	0.833	0.929	0.928	0.869	0.930	0.928
	SVM	0.688	0.721	0.741	0.591	0.735	0.697
	Random forest	0.848	0.910	0.914	0.959	0.967	0.915
	Gaussian NB	0.738	0.765	0.759	0.715	0.800	0.829
	Bernoulli NB	0.844	0.768	0.765	0.915	0.824	0.788
Feature-4	Logistic Regression	0.911	0.935	0.930	0.851	0.934	0.929
	SVM	0.926	0.832	0.729	0.827	0.727	0.677
	Random forest	0.927	0.832	0.729	0.827	0.727	0.677
	Gaussian NB	0.779	0.765	0.797	0.735	0.779	0.835
	Bernoulli NB	0.844	0.768	0.765	0.915	0.824	0.788

Host: **Vibrio**		i.i.d. model			1st order mc		
		$k=4$	$k=6$	$k=8$	$k=4$	$k=6$	$k=8$
Feature-1	Logistic Regression	0.858	0.887	0.894	0.858	0.887	0.894
	SVM	0.6362	0.6690	0.6034	0.6362	0.6690	0.6034
	Random forest	0.9361	0.9399	0.8920	0.9350	0.9379	0.8863
	Gaussian NB	0.7897	0.7828	0.7121	0.7897	0.7828	0.7121
	Bernoulli NB	0.5431	0.6224	0.6224	0.5431	0.6224	0.6224
Feature-2	Logistic Regression	0.831	0.880	0.888	0.844	0.908	0.897
	SVM	0.7845	0.8690	0.6224	0.7793	0.8397	0.5741
	Random forest	0.9350	0.9376	0.8907	0.9377	0.9295	0.8796
	Gaussian NB	0.7897	0.7862	0.5862	0.8776	0.8483	0.5862
	Bernoulli NB	0.7259	0.7086	0.5879	0.8000	0.8379	0.6017
Feature-3	Logistic Regression	0.794	0.873	0.874	0.823	0.897	0.899
	SVM	0.6034	0.5983	0.6034	0.6000	0.6034	0.5983
	Random forest	0.9232	0.9229	0.8768	0.9161	0.9446	0.8973
	Gaussian NB	0.7586	0.7552	0.8052	0.8224	0.8121	0.5862
	Bernoulli NB	0.7259	0.7086	0.5879	0.8000	0.8379	0.6017
Feature-4	Logistic Regression	0.857	0.895	0.869	0.860	0.916	0.911
	SVM	0.8431	0.8552	0.7138	0.8052	0.8121	0.5431
	Random forest	0.9349	0.9337	0.9034	0.9379	0.9490	0.8820
	Gaussian NB	0.7034	0.7397	0.8155	0.8276	0.8345	0.7034
	Bernoulli NB	0.7259	0.7086	0.5879	0.8000	0.8379	0.6017

		2nd order mc			3rd order mc		
		$k=4$	$k=6$	$k=8$	$k=4$	$k=6$	$k=8$
Feature-1	Logistic Regression	0.858	0.887	0.894	0.858	0.887	0.894
	SVM	0.6362	0.6690	0.6034	0.6362	0.6690	0.6034
	Random forest	0.9344	0.9410	0.8857	0.9343	0.9388	0.8897
	Gaussian NB	0.7897	0.7828	0.7121	0.7897	0.7828	0.7121
	Bernoulli NB	0.5431	0.6224	0.6224	0.5431	0.6224	0.6224
Feature-2	Logistic Regression	0.795	0.867	0.840	0.693	0.864	0.811
	GBR	0.7143	0.6860	0.6658	0.7056	0.7279	0.6865
	SVM	0.5793	0.8052	0.5638	0.5207	0.7190	0.5517
	Random forest	0.8973	0.9068	0.8647	0.8082	0.8786	0.8487
	Gaussian NB	0.8052	0.7310	0.5759	0.5121	0.7500	0.5862
	Bernoulli NB	0.7672	0.7776	0.5759	0.7431	0.6310	0.5448
Feature-3	Logistic Regression	0.805	0.889	0.889	0.696	0.906	0.898
	SVM	0.5914	0.6034	0.5862	0.5483	0.6034	0.5862
	Random forest	0.8926	0.9404	0.8534	0.8128	0.9226	0.8853
	Gaussian NB	0.7241	0.6966	0.5862	0.4966	0.6293	0.5862
	Bernoulli NB	0.7672	0.7776	0.5759	0.7431	0.6310	0.5448
Feature-4	Logistic Regression	0.831	0.911	0.894	0.573	0.913	0.899
	GBR	0.7338	0.7942	0.7165	0.7947	0.7207	0.6975
	SVM	0.7414	0.8052	0.5138	0.6862	0.6086	0.5000
	Random forest	0.8855	0.9337	0.8821	0.8624	0.9315	0.9031
	Gaussian NB	0.8034	0.8310	0.6914	0.4897	0.7983	0.6603
	Bernoulli NB	0.7672	0.7776	0.5759	0.7431	0.6310	0.5448

References are listed in the main paper.
